# Effect of caffeine on motility and vitality of sperm and in vitro fertilization of outbreed mouse in T6 and M16 media

**Published:** 2013-09

**Authors:** Narges Nabavi, Fatemeh Todehdehghan, Abdollhossein Shiravi

**Affiliations:** 1 *Department of Animal Science, Islamic Azad University, Damghan Branch, Damghan, Iran.*; 2 *Department of Venomous Animal and Antivenin Production, Razi Vaccine and Serum Research Institute, Karaj, Iran.*

**Keywords:** Spermatozoa, Caffeine, In-vitro fertilization, Mice, Early embryo.

## Abstract

**Background:** Caffeine increases the CAMP production that stimulates spermatozoa movement. Caffeine is also used for induction of in vitro acrosome reaction in mammalian spermatozoa, an important step in achieving fertilization.

**Objective: **The aim of this study was to assess the effect of caffeine on sperm's motility, vitality and laboratory fertilization rates in mouse in two T6 and M16 media.

**Materials and Methods:** Epididymal mouse sperms were collected and treated by caffine in T6 and M16 media and their motility and vitality rates were evaluated. The pretreated sperms were added to oocytes in T6 and M16 media with and without caffeine and fertilization rates were recorded after 24 hours incubation.

**Results: **Sperm's motility (81.7±1.67%) and vitality (88.7±1.33%) rates and percentage of fertilized oocytes (67.52±8.16%) in T6 medium plus caffeine compare to control group have increased and shown significant differences at p≤0.01. While the percentages of these parameters in M16 medium supplemented with caffeine were 68.3±6.01%, 78±6.11%, and 42.6±12.96 respectively and in comparison to control group (M16 without caffeine) have not shown significant differences.

**Conclusion:** Addition of caffeine to T6 medium promotes the sperm's motility and vitality and enhances fertilization and early in vitro development of mouse embryos.

This article extracted from M.Sc. thesis. (Narges Navabi)

## Introduction

In vitro fertilization (IVF) is a useful technique for obtaining offspring from endangered, infertile or low reproductive animals and also for evaluation of fertilization mechanisms. Improvement of effective factors and techniques related to the fertilization, make IVF more efficient and stable. The factors, such as motility, vitality and number of sperms are important parameters in ova fertilization during in vitro embryo production and may affect the rate of pre-implantation and embryo development (1). 

Acrosomal reaction (structural and metabolic changes of sperm) results in high ability and fertilization capability of sperm. Studies have reported, increasing of sperm's activity enhances the rate of sperms penetration to cumulus and zona pellucida and intact sperm membrane affect its motility that are important parameters in laboratory fertilization (2). From the first in vitro fertilization to the present, many chemical and technical changes were made to improve the laboratory fertilization, a basic and standard test for assisted reproduction (3, 4). 

Researchers have considered effective factors such as different animal species, chemical media, and physical factors affecting the fertilization processes and also invented new equipments to improve the IVF's quality (4-11). Reports have shown materials like caffeine and heparin increase fertilization rate of cattle oocytes and have positive effects on laboratory fertilization by increasing capability of spermatozoa (12-16). The present investigation was carried out to study the effect of sperm pretreatment with caffeine on fertilization and embryo development following mouse IVF. NMRI mouse is an outbreed mouse can be easily housed and bred under conventional husbandry. Optimization of in vitro culture conditions in NMRI mouse embryos is useful for human assisted reproductive technology and seems to be economical and approachable for many relevant researches in the country.

## Materials and methods

Materials and culture media used in this study were all from Sigma Company and of embryo tested grade. In this experimental-analytical study, 10 female NMRI mice of seven to eight weeks old were randomly chosen and used for oocytes collection and 20 fertile male NMRI mice for sperm preparation. Animals were kept in conventional environment's conditions, temperature of 22±2^o^C, humidity of 50±5% and fresh air of 10-12 times/hr, autoclaved straw bedding, standard diet plate from Razi Vaccine and Serum Research Institute (RVSRI) and tap water ad lib were provided. 

Work on animals was carried out according to the ISIRI 7216-2 animal ethics guidelines and approved by RVSRI ethics committee (17). For sperms preparation, male animals were sacrificed by cervical dislocation and caudal epididymises were dissected out for each set of experiment. One of the epididymises was placed in tube containing T6 plus 0.4mg/ml caffeine and other one placed in T6 without caffeine as control group, and the same manner was done for M16 medium, and the tubes were placed in 5% CO2 incubator at least for 45 minutes to 1 hour at 37^o^C for sperm's swimming up and capacitation. Microscopic methods were done for determining sperm's number, vitality and motility percentage (18). 

Vitality was assessed by eosin B (0.5% in saline). A 20 μl sample of the sperm suspension was placed on a glass slide, mixed with 7 all eosin, covered by slip and observed under a light microscope at ×400 magnifications (19). In order to study the sperm motility, sperm with quick progressive in straight paths, slow progressive in straight or not straight paths and motile in place were considered motile (18). 

The oocytes were collected from super ovulated female mice, oviducts were dissected and placed in M2 medium (sigma Co) at 37^o^C and oocytes were released by tearing the swollen ampulla with fine forceps. Oocytes were transferred to drops of T6 or M16 media containing 0.4 mg/ml caffeine and 4mg/ml BSA or without caffeine, under paraffin oil. Hundred microliters (100 ul) of processed sperms (average of 1.5×10^6^ sperm/ml) were added to oocytes drops of the same medium, and incubated in 5% CO_2_, at 37^o^C. Zygote formation and embryos growth were observed and recorded up to 24 hours.

**Statistical analysis**

The T-test was used to compare the effect of caffeine in two T6 and M16 media with their own controls, and p<0.05 were considered statistically significant. All statistical analyses were performed using the Graph Pad software (GP, for windows).

## Results

Our findings showed that mean value of sperm motility and vitality percentage in T6 medium with caffeine were 81.7±1.67 and 88.7±1.33 respectively and in T6 without caffeine (control group) were 37.2±5.30 and 47.9±3.86 which are significantly different at p≤0.01. Average value of these parameters in M16 medium plus caffeine were 68.3±6.01 and 78±6.11 and in control group (M16 without caffeine) were 61.1±6.49 and 77.4±3.22, which are not significantly different (Table I). 

The rate of sperm's motility and vitality in T6 medium with caffeine was higher than the other groups. Fertilization rate in T6 with caffeine was 67.52±8.16, that is significantly different from its control group (22.1±5.56) at p≤0.01, where the rates of this parameter in M16 medium with and without caffeine were respectively 42.6±12.96 and 33.5±6.31, and not significantly different. 

The two cell embryos rates in T6 with caffeine and without caffeine were 60±16.83 and 42.5±15.83, respectively; they are not significantly different. The percentages of two cells embryos in M16 medium with caffeine, (31.3±23.66), and without caffeine, (37.7±13.48), in comparison with their parallel values in T6 medium did not show significant differences (Table II). However the rates of oocyte fertilization and two cell embryos formation in T6 medium with caffeine were much more than the other groups.

**Table I:** Percentage of motility and vitality of mouse sperm in T6 and M16 media with and without caffeine (Mean± SE)

		**Sperm parameters**	
		**Motility (%)**	**Vitality (%)**
T6 medium			
	Control	37.2 ± 5.30	47.9 ± 3.86
	With caffeine	81.7 ± 1.67	88.7 ± 1.33
	p-value for T-test	0.0081	0.0002
M16 medium			
	Control	61.1 ± 6.49	77.4 ± 3.22
	With caffeine	68.3 ± 6.01	78 ± 6.11
p-value for T-test	0.6256	0.9333

The T-test was used for statistical analysis and p<0.05 were considered statistically significant.

**Table II:** Percentage of fertilized oocytes and two cell embryos in two T6 and M16 media with and without caffeine (Mean± SE)

		**Embryo growth stages**	
		**Fertilized oocytes (%)**	**Two cells (%)**
T6 medium			
	Control	22.1 ± 5.56	42.5 ± 15.83
	With caffeine	67.52 ± 8.16	60 ± 16.83
	p-value for T-test	0.001	0.537
M16 medium			
	Control	33.5 ± 6.31	37.7 ± 13.48
	With caffeine	42.6 ± 12.96	31.3 ± 23.66
	p-value for T-test	0.490	0.809

The T-test was used for statistical analysis and p<0.05 were considered statistically significant.

**Figure 1:**
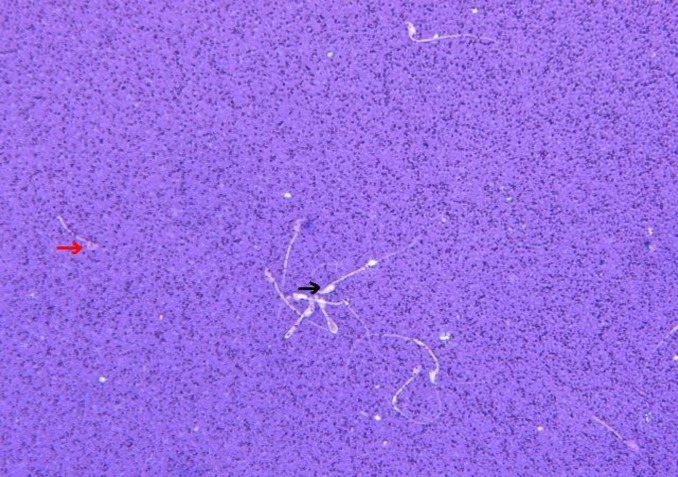
Illustration of live sperm (►) and dead sperm (►) in T6 plus caffeine (400 x).

**Figure 2:**
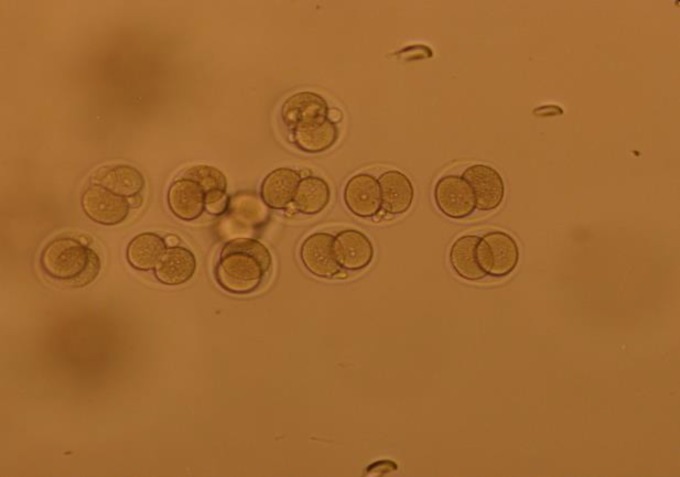
Two cell embryos of NMRI mouse, 24 hr after fertilization in T6 plus caffeine (200 x).

## Discussion

Treatment of semen with compounds such as heparin, pentoxifylline, bovine serum albumin, caffeine, dithiothreitol, ethanol, and lysophosphatidyl-choline have been used for the induction of an in vitro acrosome reaction in mammalian spermatozoa (14, 20-22). Motility, vitality rates and number of sperms are vital parameters in fertilization activity of sperms and play essential role in changing fertilization rate (16). Many researches have been done to increase ability and capability of sperms and qualitative and quantitative improvement of laboratory fertilization. In this study, the percentages of sperm's motility and fertilized oocytes in T6 plus caffeine were significantly different from T6 without caffeine that are according to Garty *et al* and Zhang and Hong's reports (23, 24).

The rate of sperms vitality in T6 medium containing caffeine as compare to T6 without caffeine has shown significant differences (16). Where, there was no significant difference in motility and vitality percentage of sperms and also number of fertilized oocytes in M16 medium containing caffeine when compared with M16 control group (without caffeine). So, it seems that factors like type and quality of culture medium have had effect on sperm parameters and their fertilization ability. It has been cleared that caffeine causes increment of CAMP inside of cell by controlling of phosphodiesterase which is analyzing enzyme of CAMP (12). 

CAMP interferes with stimulation of tyrosine phosphorylation process in spermatozoa capacitation and also, it is reported that CAMP, directly stimulates the spermatozoa movement (23, 25). The researches have shown that ROS (Reactive Oxygen Species) has important role in fertility and infertility and cause spermatozoa's hyperactivity, capacitation, acrosomal reaction and connection to zona pellucida (2). ROS production and inside flow of Ca^2+^ are first events of spermatozoa capacitation process. At the beginning of capacitating process, an unknown factor causes oxides activation in spermatozoa plasma membrane which results in necessary crop of O_2_^-^ that O_2_^-^ change to H_2_O_2_ automatically. H_2_O_2_ can activate adenylcyclase and or causes inside flow of Ca^2+^. 

Calcium can activate adenylylcyclase which in turn increases the CAMP production. However, reports regarding the success of laboratory fertilization are different, and the differences could be related to animal species, capacitation rate of sperms, genetic integrity of sperm, temperature effect and additional light at work time, increasing of time distance between hormone stimulation of animals (for animal super ovulation) and oocytes collection, culture medium quality, ovum fertilization rate, ovum accumulation in fertilization drops, work experience and laboratory equipments (1, 4-7, 9-11, 26-35). 

According to our findings, it is proposed that caffeine can be used as a supplement in culture medium for sperm collection and laboratory fertilization of mouse and T6 is a suitable medium for obtaining of larger number of high quality two cells embryos. 
